# Relative Humidity Sensor Based on No-Core Fiber Coated by Agarose-Gel Film

**DOI:** 10.3390/s17102353

**Published:** 2017-10-16

**Authors:** Wei Xu, Jia Shi, Xianchao Yang, Degang Xu, Feng Rong, Junfa Zhao, Jianquan Yao

**Affiliations:** 1 Institute of Laser and Optoelectronics, College of Precision Instrument and Optoelectronic Engineering, Tianjin University, Tianjin 300072, China; xuwei@tjpu.edu.cn (W.X.); tjushijia@tju.edu.cn (J.S.); yangxianchao@tju.edu.cn (X.Y.); xudegang@tju.edu.cn (D.X.); 2 Key Laboratory of Optoelectronic Information Science and Technology (Ministry of Education), Tianjin University, Tianjin 300072, China; 3 Tianjin Key Laboratory of Optoelectronic Detection Technology and Systems, Tianjin Polytechnic University, Tianjin 300387, China; rongfeng@tjpu.edu.cn (F.R.); zhaojunfa@tjpu.edu.cn (J.Z.)

**Keywords:** relative humidity measurement, agarose, no-core fiber, multimode interference

## Abstract

A relative humidity (RH) sensor based on single-mode–no-core–single-mode fiber (SNCS) structure is proposed and experimentally demonstrated. The agarose gel is coated on the no-core fiber (NCF) as the cladding, and multimode interference (MMI) occurs in the SNCS structure. The transmission spectrum of the sensor is modulated at different ambient relative humidities due to the tunable refractive index property of the agarose gel film. The relative humidity can be measured by the wavelength shift and intensity variation of the dip in the transmission spectra. The humidity response of the sensors, coated with different concentrations and coating numbers of the agarose solution, were experimentally investigated. The wavelength and intensity sensitivity is obtained as −149 pm/%RH and −0.075 dB/%RH in the range of 30% RH to 75% RH, respectively. The rise and fall time is tested to be 4.8 s and 7.1 s, respectively. The proposed sensor has a great potential in real-time RH monitoring.

## 1. Introduction

In recent years, various forms of optical fiber relative humidity (RH) sensors have been proposed by using side-polished fiber [1,2], photonic crystal fiber [3], tapered fiber [4,5,6], fiber Bragg gratings [7,8,9], long period gratings [10], multi-mode fibers [11,12], hollow core fiber [13], single-mode hetero-core fibers [14,15], and all kinds of interference structures [16,17,18], etc. The adsorption of thin-films on the structures mentioned above expand the traditional domains where optical fiber is used [19]. In most of the optical fiber RH sensors mentioned above, a hygroscopic material as a thin-film coating is necessary to realize the RH detection. The relative humidity can be measured indirectly through the refractive index (RI) changes of the coating material or the structure deformation induced by the hygroscopic material. A large variety of hygroscopic materials have been reported, such as agarose [3,4,17], polyvinyl alcohol [8], poly (ethylene oxide) (PEO) [14], poly (methylmethacrylate) (PMMA) [9], hydroxyethyl cellulose and polyvinylidene fluoride (HEC/PVDF) [11], hydrogel [10], graphene oxide [13], metallic oxide film [2,5,16], etc.

Based on the previous reports, the design of a optical fiber humidity sensor is basically divided into two steps. The first step is the fabrication of an optical fiber sensing platform, and the second is the deposition of a coating material. The single-mode–no-core–single-mode fiber (SNCS) structure is a good platform for the fabrication of simple and cost-effective optical fiber sensors [11,19]. For example, the SNCS structure has been applied as a real-time monitor of gasohol quality [20], as a biosensor coated by immunoglobulins (IgGs) [21], and as an chemical detector coated by zeolite [22]. The RI sensitivity of SNCS structure can be enhanced by coating a thin-film [19,23,24,25], and some special applications are achieved by coating functional materials [21,22]. According to References [19,23,24,25], the sensing sensitivity of RH can be optimized by controlling the thickness of the coating layer.

Agarose gel is widely used for RH monitoring due to the low cost, convenient fabrication, and good performance in the related reports [26]. The convenient fabrication of SNCS structure and agarose gel will reduce the difficulty in the fabrication of optical fiber humidity sensors. The effect of agarose gel coating thickness was investigated by Mathew [27], and the results showed that the RI of the agarose-gel film increased as the thickness of the film increased. The RH sensing can be theoretically achieved by coating the agarose gel film with an appropriate thickness on the SNCS structure. To accomplish this cost-effective RH sensor, the SNCS structures were coated by agarose gel with different concentrations and different coating numbers. The evolution of the transmission spectrum of the SNCS structure after the coating of hygroscopic material was also demonstrated. The results showed that the variation tendency of the transmission spectra as RH increase was just the opposite of the dehydration process during the coating.

In this paper, an agarose gel-based single-mode–no-core–single-mode optical fiber (SNCS) structure sensor for RH measurement is presented. Humidity sensors are fabricated by dip-coating technology in agarose solutions with different concentrations and coating numbers. The interesting results of the RH response of the SNCS structure coated by agarose is explained through the effective RI of the thin-film, which is decided by both the thickness and the water content. A good sensitivity of the SNCS structure coated by 0.1 wt/vol % agarose gel solution was obtained. The wavelength and intensity sensitivity is −149 pm/%RH and −0.075 dB/%RH in the range of 30% RH to 75% RH, respectively. The rise and fall times were tested to be 4.8 s and 7.1 s, respectively. The proposed sensor has a great potential in real-time RH monitoring.

## 2. Principles and Fabrication

A reproducible sensor fabrication method is established by standardizing all experimental steps including the preparation of SNCS structures and the coating process of agarose gel film (AGF).

**Figure 1:**
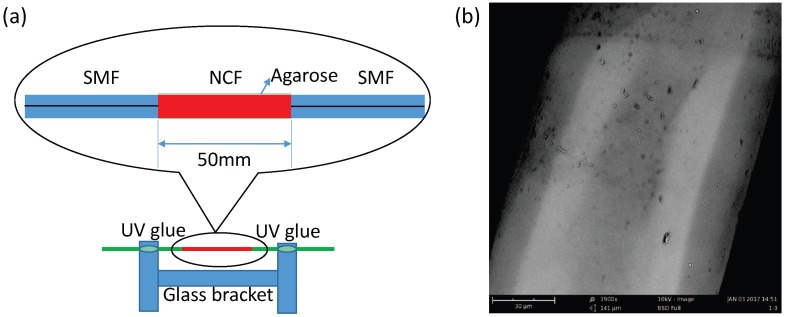
(**a**) The single-mode–no-core–single-mode fiber (SNCS) sensor fixed on an I-shaped glass bracket with UV glue and its details in the marked area; (**b**) SEM photo of the sensor coated by 0.1 wt/vol % agarose solution. SMF: single-mode fiber; NCF: no-core fiber.

As shown in Figure 1a, the SNCS structure was fixed on an I-shape glass bracket with UV glue to avoid stress variation caused by the agarose volume. When the incident light transmits from the single-mode fiber (SMF) to the no-core fiber (NCF), the high-order modes are excited and propagate within the NCF. The excited modes in the NCF interfere with one another as they propagate along the whole length of the NCF, which is called multimode interference (MMI). Because the effective refractive index (ERI) of the AGF changes with the ambient RH, the transmission spectra vary accordingly. Therefore, it can be used as an RH sensor. The length and diameter of the NCF (Tianjin Opticontact Technology Development Co., Ltd., Tianjin, China) used in this experiment were about 53.5 mm and 100 μm, respectively. The SMF had a step index profile with core/cladding diameters of 8.2/125 μm. The structure was fabricated by a commercial fusion splicer (FSM-60s, Fujikura (Chinese) Co. Ltd., Beijing, China). During the experiment, the optical spectrum analyzer (OSA, AQ6370B, Yokogawa China Co., Ltd., Shanghai, China) were operated at the resolution of 0.1 nm in High1 mode, and the power of the super luminescent diode (SLED) (C + L band, Zewda Technology Co., Ltd., Shenzhen, China) was set at 10 mW.

The next step in the sensor fabrication was coating the SNCS structure with the hydrophilic agarose gel. The 0.05 wt/vol % and 0.1 wt/vol % agarose solution was prepared. The agarose powder (Biowest Regular Agarose G-10) was dissolved in water, then the solutions were heated to boiling with an electromagnetic oven. To dissolve the agarose in distilled water, the beaker containing the mixture was placed on a magnetic stirrer for ten minutes. The SNCS structure samples were dipped into the hot agarose solution and pulled out after 2 s. In order to analyze the coating process, the transmission spectra in the coating process were recorded by the OSA.

**Figure 2:**
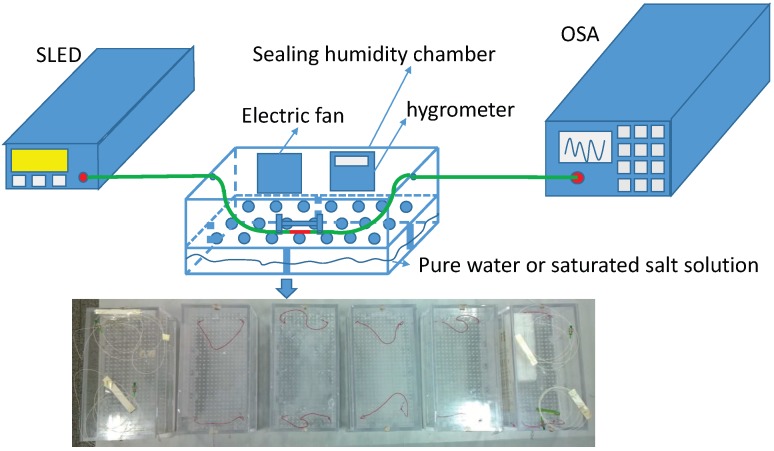
Schematic diagram of the experimental setup for RH measurement. OSA: optical spectrum analyzer; SLED: super luminescent diode.

This study adopted a very convenient way to measure and calibrate humidity sensors by using saturated salt solutions. As shown in Figure 2, the saturated salt solution, which was made up of a slushy mixture with distilled water and chemically pure salt, was enclosed in a customized sealable organic glass chamber (six chambers were customized) to build mini-environments with different RH values. The RH of the atmosphere above the saturated salt solution in the sealed chamber was fixed at a given temperature, and it could cover almost the entire range of relative humidity, as shown in Table 1. Because the concentration of a saturated salt solution is fixed at a given temperature, it is very easy to determine the saturation state when there is solid phase salt left in the solution. Due to the limited fixed RH points, we firstly calibrated a standard electric hygrometer in five fixed humidity chambers with the saturated salt solutions, and then used a chamber with pure water to test the sensors which were placed with the calibrated hygrometer. It can theoretically reach 100%RH in the sealed chamber with pure water. To speed up the calibration process, we used an electric mini-fan in the mini-environment to speed up the ambient humidity to its fixed values. Aschematic diagram of the experimental setup for humidity measurement is shown in Figure 2.

## 3. Experimental Results and Discussion

**Figure 3:**
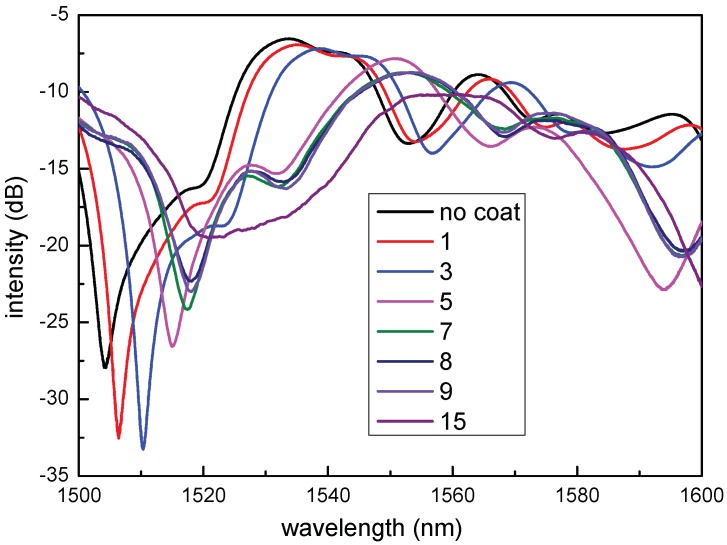
Transmission spectra of the sensor coated by 0.05 wt/vol % agarose solution in different coating numbers.

**Figure 4:**
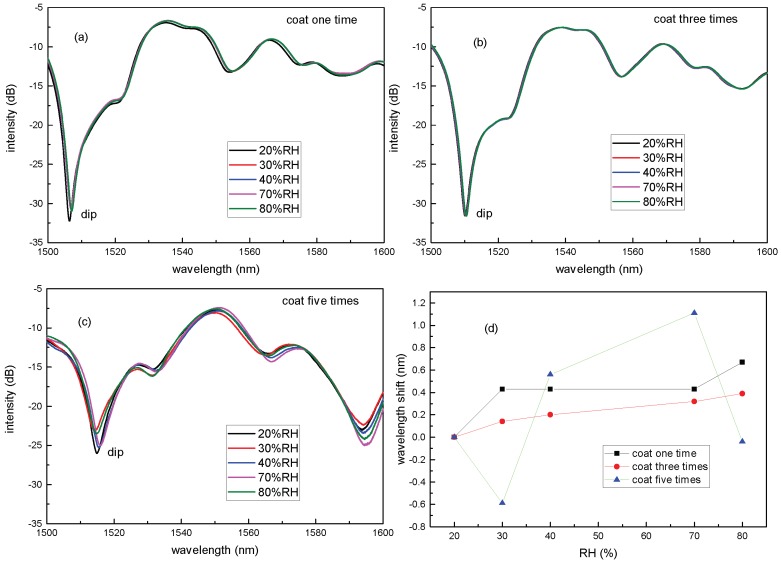
Transmission spectra at different RH values of the sensor coated by 0.05 wt/vol % agarose mixture in different numbers: (**a**) coat one time; (**b**) coat three times; (**c**) coat five times. (**d**) The wavelength shift of the dip in different RH.

Firstly, the SNCS structure was coated by 0.05 wt/vol % agarose solution with different coating numbers. It should be noted that each coating was carried out until it dried after the last coating. The transmission spectra of the SNCS structure with different coating numbers are shown in Figure 3. There were red shifts in the spectra as the coating number increased. This means that the ERI of the AGF increased with the coating numbers. It is obvious that the thickness of the coating increased when the coating number increased. It is consistent with the fact that the refractive index of the AGF experienced by the mode interacting with the AGF depends on the thickness of the coating [27]. However, with more than 15 times coating, the transmission spectrum of the SNCS structure disappeared. This may be caused by the fact that the RI of the coating is higher than the RI of the NCF and the guided modes in the NCF are all leaked to the coating. The transmission spectra response to RH of the sensors coated in different numbers are shown in Figure 4. There were tiny red shifts and intensity transfer in the spectra of the sensors with one and three times coating, as shown in Figure 4a,b. In Figure 4c, there is a blue shift between 20% RH to 30% RH, a red shift between 30% RH to 70% RH, and a blue shift between 70% RH to 80% RH. The positive and negative sensitivity values represent spectral shift in opposite directions, which may be caused by the complicated variation of the AGF ERI response to RH [27]. When the RH rises, more water molecules are diffused into the agarose film, resulting in its inflation and increment of its thickness. The increment of the thickness [27] and the substitution of water molecules to air holes [28] in the AGF lead to the increment of its ERI. Similar to any other swelling polymer, the increase of water content will decrease the refractive index of the agarose coating according to the Lorenz–Lorentz relation. The total ERI is decided by the interaction of these two cases. Whichever case is dominant, the ERI changes in that case. In one and three times coating, the decrement of the ERI may be weaker than the increment, so the total ERI is dominated by increment. In five times coating, the complex spectral shift of the sensor can be explained by that the dominant effect is changing in different humidity ranges. Therefore, in order to achieve a monotonic response to ambient RH, the coating numbers using 0.05 wt/vol % agarose solution should be less than five in the experiment. However, the low sensitivity limits the application.

**Figure 5:**
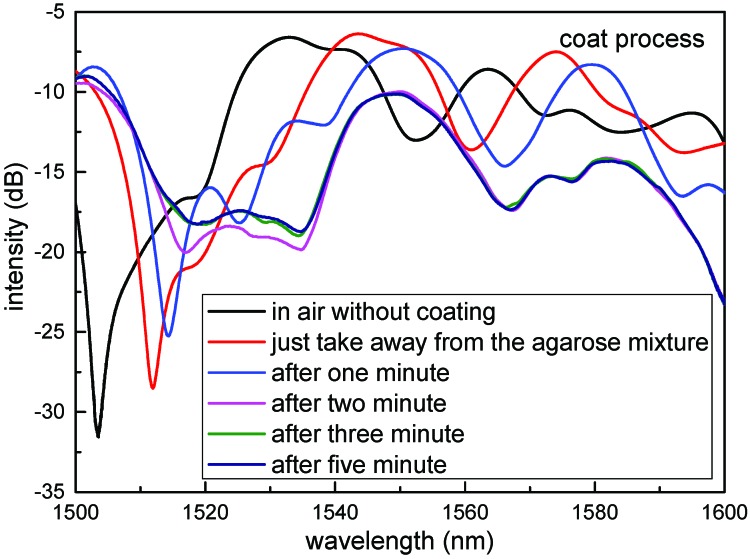
Transmission spectra of the sensor coated in one time by 0.1 wt/vol % agarose solution when the agarose gel film (AGF) is dehydration with time goes on.

**Figure 6:**
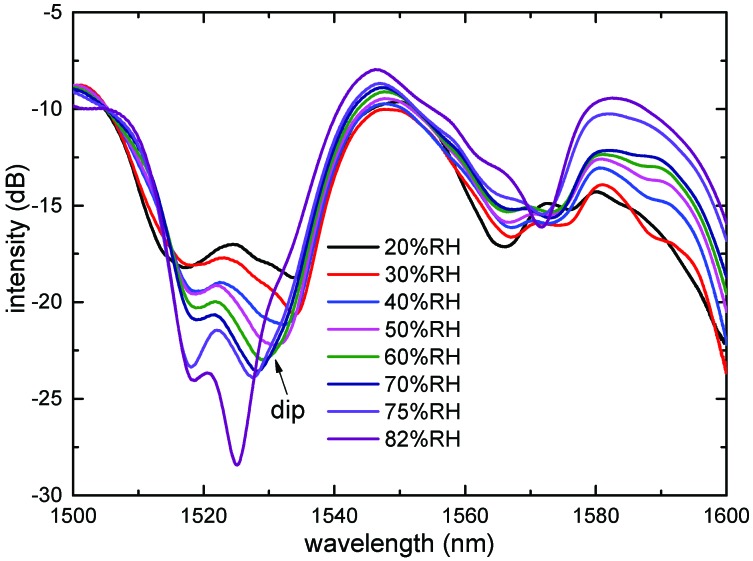
Transmission spectra at different RH values of the sensor coated by 0.1 wt/vol % agarose solution.

**Figure 7:**
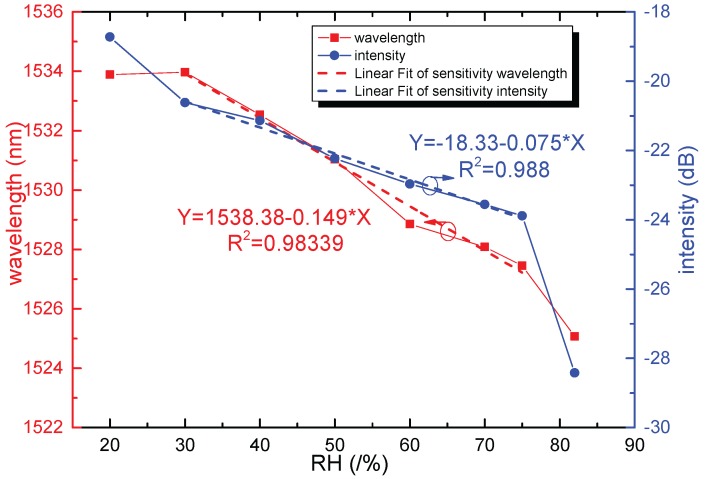
The dependence of the dip wavelength and intensity of the sensor coated by 0.1. wt/vol % in RH range from 30%RH to 75%RH.

Another SNCS structure coated by 0.1 wt/vol % agarose solution was fabricated. The SEM photo of the SNCS structure coated with one time by 0.1 wt/vol % agarose solution is shown in Figure 1b. The thickness of the AGF was about 1 μm. It should be noted that the coating process and the SEM photo were completed at room temperature of 25 °C and relative humidity of 20%RH. As shown in Figure 5, the spectra of the sensor with coated one time have a red shift when the AGF is dehydration with times goes on. The transmission spectrum changed obviously during the formation of the agarose coating. The final transmission spectrum coated with one time by 0.1 wt/vol % was similar to the transmission spectrum coated by 0.05 wt/vol % with 15 times. The ERI of the AGF may be close to the NCF in the process of dehydration, and some high guided modes have become leaky modes. The transmission spectra of the SNCS structure coated by 0.1 wt/vol % agarose solution at different RH values are measured in Figure 6. With the increase of RH, the variation tendency of the transmission spectra of this structure is just the opposite of the dehydration process during the coating. The RH response can be predicted through the transmission spectra in the coating process. The dip had a blue shift of 6.7 nm in the range of 30% RH to 75% RH, and the corresponding changes of dip intensity are −3.27 dB. The dependence of the dip wavelength and intensity on RH range from 20% RH to 82% RH are shown in Figure 7. The wavelength and intensity sensitivity was −149 pm/%RH and −0.075 dB/%RH in the range of 30% RH to 75% RH, respectively. It is difficult to obtain a large measurement range and linearity by coating agarose gel because the change trend of the ERI is not in one direction with the RH. However, we can select the appropriate measurement range according to the actual application.

**Figure 8:**
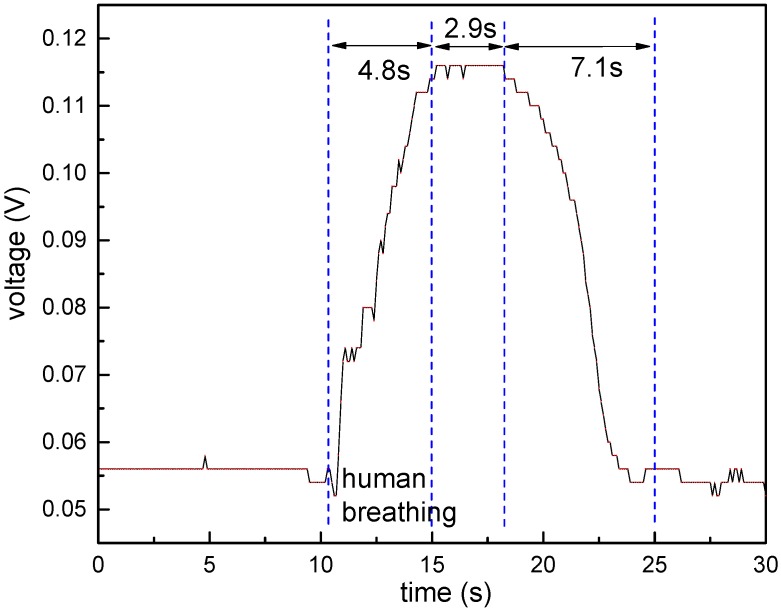
The voltage response of the sensor coated by 0.1 wt/vol % agarose solution.

In order to analyze the response time, a tunable semiconductor laser (TSL-510, Santec (Shanghai) Co. Ltd., Shanghai, China) and a high-speed PIN photodetector (wavelength: 1020–1650 nm, frequency: 0–3 GHz) were connected by the sensor coated by 0.1 wt/vol % agarose solution. The commercial humidity chamber was not suitable for studying the response time of the sensor because of the slow adjustment. Similar to [18], a human breathing was applied directly on the fiber for about 5 s. The response time is shown in Figure 8. The voltage value of the photodetector (there is a reverse amplification) at the wavelength of 1520 nm reached sharply 115 mV, and remained at 115 mV for about 2.9 s. After that, the voltage value decreased to about 55 mV and remained stable. It should be noted that accurate output voltage can be obtained by using a high-sensitivity analog-to-digital converter, but more voltage fluctuations will be acquired, and complex digital filtering techniques are needed. The rise time of the sensor was about 4.8 s. The recovery time was about 7.1 s, which depends on the time of the water vapor removed from the agarose.

## 4. Conclusions

As mentioned above, due to the complicated RI response of agarose film to RH, it is difficult to obtain a large measurement range and linearity by coating with agarose gel. However, we can select the appropriate measurement range according to the actual application. In our experiments, the wavelength and intensity sensitivity is obtained as −149 pm/%RH and −0.075 dB/%RH in the range of 30% RH to 75% RH. The rise and fall time is tested to be 4.8 s and 7.1 s, respectively. The proposed sensor has a great potential in real-time RH monitoring.

## Figures and Tables

**Table 1:** Relative humidity (RH) values of normally used saturated salt solutions.

Saturated Salt Solution	Temperature (°C)	RH (%)
MgCl	25	33
K_2_CO_3_	25	43
NaCl	25	75
KCl	25	87
K_2_SO_4_	25	98
